# Effect of Corn Particle Size on the Particle Size of Intestinal Digesta or Feces and Nutrient Digestibility of Corn–Soybean Meal Diets for Growing Pigs

**DOI:** 10.3390/ani10050876

**Published:** 2020-05-18

**Authors:** Qingtao Gao, Feng Zhao, Fangkun Dang, Hu Zhang, Ya Wang

**Affiliations:** The State Key Laboratory of Animal Nutrition, Institute of Animal Sciences, Chinese Academy of Agricultural Sciences, Beijing 100193, China; qingtao_gao@163.com (Q.G.); dangfangkun@163.com (F.D.); ndzhanghu@163.com (H.Z.); wangya920708@163.com (Y.W.)

**Keywords:** corn particle size, digesta particle size, nutrients digestibility, growing pigs

## Abstract

**Simple Summary:**

Dietary particle size can affect the digestible or metabolizable energy values of diet for pig. So, the mean particle size (MPS) of corn-soybean meal diet was recommended to less than 600 µm to improve growth performance and efficiency of dietary nutrients utilization by growing pigs. However, little information is available on whether further reduction of MPS influences the digestion process and increases digestibility of nutrients. This study evaluated the effects of reducing particle size of corn from 682 to 365 µm on the particle size of intestinal digesta or feces and nutrient digestibility of corn-soybean meal diets for growing pigs. The results showed that the MPS of duodenal digesta, ileal digesta and feces was reduced with the reduction of corn particle size. But, the MPS of corn did not affect the activities of amylase, trypsin and chymotrypsin in the duodenal fluid and the apparent total tract digestibility (ATTD) of dry matter, gross energy, crude protein, ether extract, neutral (NDF) and acid detergent fiber (ADF). These results suggest that the MPS less than 511 µm for corn-soybean meal diet does not improve the utilization of nutrients, thus it’s not necessary to further reduce particle size of corn with MPS of 682 µm.

**Abstract:**

This study was conducted to evaluate the effect of corn particle size on the particle size of intestinal digesta or feces and nutrient digestibility of corn–soybean meal diets. Twenty-four growing barrows (initial BW: 21.9 ± 1.62 kg) were randomly divided into 4 groups of 6 pigs. A T-cannula was surgically placed in the anterior duodenum (about 50 cm from pylorus) of pigs in Groups 1 and 2 or in the distal ileum of pigs in Groups 3 and 4. Corn used to formulate diets had mean particle size (MPS) of 365 µm (Corn 1) or 682 µm (Corn 2), resulting in diets with MPS of 390 µm (Diet 1) or 511 μm (Diet 2). Diet 1 or 2 were randomly assigned within pig Groups 1 or 2 and 3 or 4. The digestive enzyme activities of duodenal fluid, particle size of intestinal digesta and feces, as well as nutrient digestibility, were determined for each pig as the experiment unit. The MPS of duodenal digesta (181 vs. 287 µm, *p* < 0.01), ileal digesta (253 vs. 331 µm, *p* < 0.01), and feces (195 vs. 293 µm, *p* < 0.01) was significantly reduced for pigs fed Diet 1 vs. Diet 2, respectively. Compared with Diet 2, Diet 1 significantly reduced the proportion of particles above 0.5 mm, but significantly increased the proportion of particles between 0.072 and 0.5 mm (*p* < 0.01) in digesta and feces (*p* < 0.01). Diet 1 significantly increased solubles percentage (<0.072 mm) in duodenal digesta (*p* < 0.05) but did not affect solubles percentage in ileal digesta and feces. The MPS of diet did not affect the activities of amylase, trypsin, and chymotrypsin in the duodenal fluid and the apparent total tract digestibility (ATTD) of dry matter, gross energy, crude protein, ether extract, neutral detergent fiber (NDF) and acid detergent fiber (ADF) in pigs offered Diet 1 compared to Diet 2. The in vitro digestible energy (IVDE) (3706 vs. 3641 kcal/kg; *p* = 0.03) was greater for Corn 1 vs. Corn 2. However, no significant difference was observed in IVDE (3574 vs. 3561 kcal/kg; *p* = 0.47) for Diet 1 vs. Diet 2. In conclusion, the particle size of digesta and feces was dependent on the dietary particle size. However, the digestive enzyme activities of duodenal fluid and ATTD of energy and nutrients were not affected by reducing dietary MPS from 511 to 390 µm.

## 1. Introduction

Corn is the primary energy feed ingredient for pigs in China. Its digestible or metabolizable energy depends on chemical composition and particle size [1,2,3,4]. Previous studies with nursery and finishing pigs indicate that the efficiency of gain improves with reduced corn particle size [5,6,7]. Amaral et al. [8] observed that the energy digestibility of corn fed to growing pigs was increased because of increased digestibility of starch when the particle size was reduced from 850 to 550 µm. The results of Rojas and Stein [2] have shown that reducing particle size from 865 to 339 µm linearly increased the energy digestibility of corn. Reducing mean particle size (MPS) of corn–soybean meal diets below 500 to 600 µm is generally reported to have a positive effect on nutrient and energy digestibility [2,9]. However, results related to the energy digestibility of corn with MPS below 500 µm are variable and inconsistent in several studies [4,10,11]. Corn used to formulate diets for growing-finishing pigs in China is generally ground pass through 2 to 2.5 mm sieves with MPS of 618 to 659 µm [4]. There is little information to clarify whether corn particle size reduced to less than 500 µm is more valuable to fed pigs. Furthermore, studies have focused on the relationship between particle size and nutrient digestibility [12,13], but did not evaluate the impact of dietary particle size on the digestion process of pigs. The objective of this experiment is to evaluate the effects of corn particle size on the enzyme activities of duodenal fluid, particle size of digesta or feces, and nutrient digestibility when the pigs are fed a diet of corn ground passed through a 1 or 2 mm sieve with the MPS of 365 or 682 µm.

## 2. Materials and Methods

The protocol for this experiment was reviewed and approved by the Animal Care and Use Committee of the Institute of Animal Sciences, Chinese Academy of Agricultural Sciences (Ethical code: IAS2017-5).

### 2.1. Experimental Design, Animals, Housing, and Diets

Twenty-four growing barrows (initial BW: 21.9 ± 1.62 kg) were randomly divided into 4 groups of 6 pigs. A T-cannula was surgically placed in the anterior duodenum (about 50 cm from pylorus) of pigs in Groups 1 and 2 or in the distal ileum of pigs in Groups 3 and 4 according to the procedure described by Dang [14]. Pigs were housed in individual crates in an environmentally controlled room. Each crate had smooth and plastic-coated sides, a slatted floor, a feeder, and a nipple drinker. Pigs were allowed to recover from surgery for 14 d. Diets 1 or 2 were randomly assigned within pig Groups 1 or 2 and 3 or 4.

Diets were formulated with corn ground to pass through 1 mm (Diet 1) or 2 mm (Diet 2) sieve, and soybean meal ground to pass through a 1.5-mm sieve, as well as other ingredients to produce two diets with different particle sizes, then pelleted using steam conditioning. Dietary nutrient contents were formulated to meet the nutrient requirements for growing pigs [15] (Table 1).

### 2.2. Feeding and Sample Collection

Pigs were provided with ad libitum access to water and were fed a daily amount of diet equivalent to 4% of BW (29.1 ±1.58 kg) at the beginning of the experiment. The amount was split into two equal meals offered at 08:00 and 16:00.

After 5 days of adaptation, total feces were collected from each pig on days 6 to 10. Feces were immediately stored at −20 °C after each collection to later determine the apparent total tract digestibility (ATTD) of nutrients. On days 11, 13 and 15, duodenal digesta was collected by T-cannula twice daily for 1 h per day with each beginning at 09:00 and 17:00, and ileal digesta was collected 3 times a day for 1 h with each beginning at 09:00, 12:00, and 17:00. Digesta was centrifuged (10 min at 4 °C, 1250× *g*) to separate intestinal fluid from the digesta residues, according to the procedure described by Zhao et al. [16]. Samples were stored at −20 °C. Feces of each pig were collected on days 12 and 14 to determine the particle size. Samples were thawed and pooled between pigs after completion of the trial. Fecal samples were dried at 65 °C in a forced-air oven for 72 h prior to analysis for nutrients.

### 2.3. Determination of Particle Size, Digestive Enzyme Activities and Digestibility

The particle size of pelleted diets, digesta, and feces was determined by wet-sieve techniques described by Lentle et al. [17] and Gao et al. [18]. In brief, 7.5 to 10 g of pelleted diets, 15 to 30 g of digesta residues, or 10 to 20 g of feces were weighed and suspended in 100 mL of distilled water for 15 min prior to sieving and washed through a nest of sieves (GB/T 2006) of decreasing size (2, 1, 0.5, 0.25, 0.106 and 0.071 mm opening diameter) for 4 min with a vibration frequency of 90 to 100 times per min. The contents on each sieve were washed onto a dried, pre-weighed tinfoil box, subsequently dried at 65 °C for 24 h, then dried at 105 °C for 4 h in a forced-air oven. The weight of particles from each sieve was expressed as a percentage of total dry matter (DM).

The frozen duodenal fluid samples were thawed in water at 4 °C before determining digestive enzyme activities. *α*-amylase was measured with soluble starch as a substrate according to procedures described by Dahlqvist [19]. Trypsin was measured using Na-*p*-toluolsulfonyl-L-arginine methyl ester hydrochloride as a substrate. Chymotrypsin was measured using N-benzoyl-L-tyrosine ethyl ester as a substrate according to procedures described by Wirnt [20,21].

The in vivo digestibility of dietary nutrients was determined according to the method described by Kong and Adeola [22]. The in vitro digestibility of DM (IVDDM) and GE (IVDGE) as well as in vitro digestible energy (IVDE) of corn and diets were determined using a computer-controlled simulated digestion system with 3, 5 and 21 h for gastric, small intestinal, and large intestinal digestion according to the procedure described by Gao [23].

### 2.4. Chemical Analysis for Diet, Feces, and Residue

Diets or feces were ground to pass through a 0.42-mm sieve and thoroughly mixed before analysis. Diets and feces were analyzed for DM (method 930.15; AOAC [24]), CP (method 984.13; AOAC [24]), ether extract (EE; method 920.39; AOAC [24]), neutral (NDF) [25] and acid detergent fiber (ADF; method 973.18; AOAC [24]). GE of diets, feces or in vitro undigestible residue was measured using an automatic isoperibol oxygen bomb calorimeter (Parr 6400 Calorimeter; Parr Instrument Company, Moline, IL, USA).

### 2.5. Calculations and Statistical Analysis

Mass of particles retained on each sieve was expressed as P_i_ (the proportion of particles on each sieve in total DM). The MPS was calculated according to the formula described by Clauss et al. [26].

MPS = ∑ [P_i_ × (S_i_ + S_i + 1_)/2] (1)

Sieves were ordered from minimum up to maximum pore size. S_i_ and S_i + 1_ were the dimension of the i-size sieve and the next larger sieve.

The in vivo digestibility of nutrients and DE (kcal/kg of DM) in diet was calculated using the following equations [22,27]:

Digestibility (%) = (C*_input_* − C*_output_*)/C*_input_* × 100 (2)

where C*_input_* and C*_output_* are the amounts of component ingested and voided via the feces, respectively.

DE (kcal/kg DM) = (GE*_I_ −* GE*_F_*)/DMI (3)

in which GE*_I_* and GE*_F_* are GE intake and output in feces (kcal/d), respectively; DMI is DM intake (kg/d).

The IVDDM, IVDGE, and IVDE were calculated using the following equations [28]:

IVDDM (%) = (sample DM weight − defatted residue DM weight)/sample DM weight(4)

IVDE = [(sample DM weight × sample GE) − (defatted residue DM weight × defatted residue GE)]/sample DM weight(5)

IVDGE (%) = IVDE/sample GE(6)

Outliers (observation deviated between 1.5 times interquartile below 25th percentile and above 75th percentile) were detected using the BOXPLOT procedure of SAS (version 9.0; SAS Institute Inc. Cary, NC, USA) and removed from the data set [29]. Data were analyzed as a two-sample comparison with the pig as the experimental unit. Treatment means were calculated using the MEANS procedure of SAS (version 9.0; SAS Institute Inc. Cary, NC, USA). Differences among the particle size in diets, digesta or feces, digestive enzyme activity, ATTD, in vitro digestibility of DM or GE, and IVDE in the two diet treatments or two corns were detected using the TTEST procedure of SAS (version 9.0; SAS Institute Inc. Cary, NC, USA). Statistical differences were established at *p* ≤ 0.05, whereas 0.05 < *p* ≤ 0.1 was considered a trend.

## 3. Results

### 3.1. Particle Size of Corn, Diets, Digesta, and Feces

The MPS calculated from the distribution of particle size was 365 or 682 µm for Corns 1 or 2 and 390 or 511 µm for Diets 1 or 2, respectively. Corn 1 contained fewer particles from 1 to 2 mm but more particles from 0.106 to 1 mm and <0.072 mm. Diet 1 had fewer particles from 0.5 to 2 mm and more particles from 0.106 to 0.5 mm than Diet 2 (Table 2). However, the distributions of particles from 0.072 to 0.106 mm and <0.072 mm did not differ across diets.

The MPS was calculated 181 vs. 287 µm for duodenal digesta (*p* < 0.01), 253 vs. 331 µm for ileal digesta (*p* < 0.01), and 195 vs. 293 µm for feces (*p* < 0.01) for pigs fed Diet 1 vs. Diet 2, respectively (Table 3). Diet 1 resulted in fewer particles from 0.5 to 2 mm in duodenal and ileal digesta and feces (*p* < 0.01), but increased particles from 0.072 to 0.5 mm (*p* < 0.01) in contrast to Diet 2. Diet 1 increased particle size <0.072 mm in duodenal digesta (*p* = 0.04), but there was no effect of diet on the particle size <0.072 mm in ileal digesta and feces.

### 3.2. Digestive Enzyme Activity in Duodenal Fluid and ATTD of Nutrients in Diets

The activities of digestive enzymes in the duodenal fluid were 147.7 vs. 137.6 U/mL for amylase, 43.6 vs. 46.0 U/mL for trypsin, and 4.7 vs. 7.2 U/mL for chymotrypsin in pigs fed Diet 1 vs. Diet 2, respectively (*p* > 0.05) (Table 4). No statistically significant difference in feed intake was observed for two diets (data not shown). The values of ATTD were 88.6 vs. 88.2% for DM, 86.6 vs. 86.4% for CP, 84.6 vs. 83.7% for EE, 60.3 vs. 57.9% for NDF, 54.7 vs. 59.5% for ADF, 88.8 vs. 88.5% for GE, and 3970 vs. 3953kcal/kg for DE in pigs fed Diet 1 vs. Diet 2, respectively (*p* > 0.1).

### 3.3. In Vitro Digestibility of DM, GE, and IVDE in Corn and Diets

The IVDDM did not differ across the 2 corn samples evaluated (82.5% vs. 82.3%; *p* = 0.52), but the IVDGE (82.7% vs. 81.5%; *p* = 0.07), and IVDE (3706 vs. 3641 kcal/kg; *p* = 0.03) were greater for Corn 1 compared with Corn 2, respectively (Table 5). Accordingly, the values were 81.5% vs. 81.2% for IVDDM, 81.0% vs. 80.6% for IVDGE, and 3574 vs. 3561 kcal/kg for IVDE for Diet 1 compared with Diet 2, respectively. No statistically significant differences in IVDGE or IVDE were observed for Diets 1 and 2.

## 4. Discussion

When the corn was ground and passed through 2-mm and 1-mm sieves, the MPS was reduced from 682 to 365 µm, because the 1-mm sieve reduced the proportion of particles above 1 mm, but increased the proportion of particles from 0.106 to 1 mm and <0.072 mm contrast to the 2-mm sieve. This phenomenon is consistent with the findings of Saqui-Salces et al. [13], who reported that fine ground reduced coarser particles and increased finer particles to reduce the MPS. In the current study, all ingredients were identical except the MPS of corn. Consequently, corn with MPS of 365 or 682 µm produces to Diets 1 or 2 with MPS of 390 or 511 µm, respectively. However, the difference of MPS between Corn 1 and Diet 1, Corn 2 and Diet 2 were 25 and 171 µm, respectively. It is due to steam pelleting, which may further reduce the MPS of diet [11], and there might be a positive relationship between the MPS of diet and the extent of MPS change from pelleting.

During digestion, dietary nutrients are gradually digested from macromolecules to small molecules. Thus, it might be deduced that the particle size of digesta should gradually decrease from the duodenum, ileum to feces. However, in the two diets of this study, the MPS of duodenal digesta was similar to that of feces but less than that of ileal digesta. A large amount of pancreatic juice and bile are secreted into the duodenum, which concentrates these secretions in the digesta, thereby increasing the proportion of the particle size less than 0.072 mm. Wilfart et al. [30] reported that the duodenal digestibility of dietary nutrients was very low and even negative due to large secretion of pancreatic juice and bile in growing pigs.

Dietary particle size can influence retention time of digesta in the gastrointestinal tract. Retention time of digesta in digestive tract increases with dietary coarseness [31,32]. This enables longer contact time for coarse particles with digestive fluid, so the extent of particle size reduction in digesta may be greater for coarse particle relative to finer grinds. In the two diet treatments of this study, the difference of MPS is 121 µm for diet, 106 µm for duodenal digesta, 78 µm for ileal digesta, and 98 µm for feces, respectively. Overall, the difference of MPS in digesta of pigs fed coarse (Diet 2) and fine (Diet 1) diets decreased along the digestive tract. This finding confirms the diameter of the large particles is reduced to a greater extent than small particles during digestion, and others have reported similar findings [33,34].

The MPS of diet directly affects the particle size of digesta. In the current study, the MPS of Diet 1 was smaller than that of Diet 2, thus the particle size of duodenal, ileal digesta and feces from Diet 1 was smaller than these from Diet 2. This phenomenon is similar to the effect of dietary particle size on digesta and fecal particle size in broiler chickens [17]. The results of Jankowski et al. [33] showed the MPS of digesta from small intestine increased with increasing proportions of whole-particle wheat.

Reduction of dietary particle size reduces retention time of digesta in gastrointestinal tract of pigs [31], which may decline the secretion of digestive enzyme [11]. This result suggests that digestive enzyme activities are affected by dietary MPS. However, the current study indicates that the activities of amylase, trypsin, and chymotrypsin in duodenal fluid are not related to MPS. This lack of congruence may relate to the fact that secretion of digestive enzymes is excessive relative to the dietary substrate for growing pigs. Furthermore, the amount of digesta flowing through the duodenum varies largely [35], so it may be difficult to conclude changes in duodenal enzyme activities from a statistical standpoint [14,36]. Similar studies have also shown that the change of dietary nutrient concentration does not affect the activities of jejunal and pancreatic enzymes for growing pigs [37,38]. The MPS of the two diets differed by only 121 µm, which was far lower than the difference (more than 400 µm) in the study described by Wondra et al. [39] and Kim et al. [40]. Such small differences of particle size may not be sufficient to cause differences in digestive enzyme activities in growing pigs.

The smaller dietary MPS increases the surface area of digestive enzymes in contact with the substrate, which is helpful to improve the digestibility of nutrients. However, others indicate that MPS is positively related to retention time of digesta [11,32], and finer MPS reduces the secretion of digestive juice so that the activities of digestive enzymes decrease. Together, these changes in digestive physiological process may not improve dietary nutrient digestibility. As a result, there may be a threshold range of MPS, below which the nutrient digestibility tends to be stable. In the current study, increment in the MPS of diets from 390 to 511 µm did not affect the in vivo ATTD of GE, CP, EE, and fiber. Conversely, reducing MPS positively affected the IVDGE and IVDE in the corn, but no statistical difference was observed on the IVDGE and IVDE between two diets with different MPS. Wondra et al. [39] observed that the ATTD of GE in corn increases by approx. 6% when it was ground to MPS from 826 to 415 µm. However, Rojas and Stein [2] reported that ATTD of GE was increased approx. 1% when the corn ground to MPS from 485 to 339 µm. This indicates that effect of MPS on nutrient digestibility of corn is greater in MPS over about 500 µm than below about 500 µm. Similarly, in other cereals and their byproducts, Bao et al. [41] observed that the ATTD of DM and GE of wheat diet increased when the MPS decreased from 670 to 430 µm, but these values were both declined when the MPS further decreased to 330 µm. Paulk et al. [42] and Healy et al. [5] reported that reduction of MPS of sorghum from 800 to 400 µm linearly increased the ratio of gain to feed in nursery or finishing pigs. Liu et al. [43] demonstrated the dietary ATTD of DM or GE increased by 1.07% and 0.44% or 1.11% and 0.73% when the MPS of distiller-dried grains with solubles in diets decreased from 818 to 594 µm and from 594 to 308 µm, respectively. The current study indicates the effect of further reduction of MPS less than 511 µm for corn–soybean meal diet on improvement of nutrients digestibility becomes smaller, thus it is not necessary to further reduce particle size of corn with MPS of 682 µm.

## 5. Conclusions

The particle size of corn positively affected the particle size of duodenal and ileal digesta and feces in growing pigs. However, the digestive enzyme activities of duodenal fluid and ATTD of dietary nutrients was not affected by dietary MPS ranged from 390 to 511 µm.

## Figures and Tables

**Table 1:** Ingredients and chemical composition of the experimental diets (as-fed basis).

Items	Diet 1	Diet 2
Ingredients, (g/kg)		
Corn 1 (ground pass through 1 mm sieve)	666.9	-
Corn 2 (ground pass through 2 mm sieve)	-	666.9
Soybean meal (ground pass through 1.5 mm sieve)	222.0	222.0
Soybean oil	11.8	11.8
Wheat flour	50.0	50.0
Limestone	7.5	7.5
Dicalcium phosphate	13.4	13.4
Sodium chloride	3.7	3.7
L-lysine sulfate	8.6	8.6
Choline chloride	0.8	0.8
Tryptophan	0.3	0.3
Threonine	2.3	2.3
Methionine	2.3	2.3
Fungicide	0.4	0.4
Vitamin and mineral premix ^a^	10.0	10.0
Analyzed nutrient contents ^b^		
GE, kcal/kg	3981	3979
DM, %	89.1	89.1
CP, %	17.3	17.1
EE, %	3.7	3.9
ADF, %	3.3	3.0
NDF, %	11.9	11.2

^a^ Vitamin–mineral premix composition (per kg feed): Vitamin: A, 12800 IU; D_3_, 4400 IU; E, 100 mg; K_3_, 4 mg; B_1_, 4 mg; B_2_, 10 mg; B_12_, 48 μg; D-pantothenic acid, 40 mg; nicotinic acid, 6 mg; folic acid, 24 mg; biotin, 2 mg. Minerals: copper, 10 mg (as copper sulfate); iron, 110 mg (as ferrous sulfate); zinc, 40 mg (as zinc sulfate); manganese, 25 mg (as manganese sulfate); selenium, 0.3 mg (as sodium selenite); iodine, 0.3 mg (as potassium iodide). ^b^ ADF, acid detergent fiber; CP, crude protein; DM, dry matter; EE, ether extract; GE, gross energy; NDF, neutral detergent fiber.

**Table 2:** The particle size classes and MPS of corn and diets ^a^.

Item	Proportion of Particle Size Classes, %						MPS ^b^, µm
	1–2 mm	0.5–1 mm	0.25–0.5 mm	0.106–0.25 mm	0.072–0.106 mm	<0.072 mm	
Corn							
1	0.86	27.40	28.33	15.32	4.79	23.30	365
2	28.23	23.73	13.65	10.40	3.48	20.51	682
Diet							
1	7.17	17.57	30.21	13.52	3.19	28.33	390
2	13.28	27.98	19.68	8.94	2.98	27.14	511

^a^ Values are duplicate measurements using the wet-sieving method. ^b^ MPS, mean particle size.

**Table 3:** Effect of dietary particle size on the proportion of particle size classes in the digesta and feces.

Item	Proportion of Particle Size Classes, %						MPS, µm
	1–2 mm	0.5–1 mm	0.25–0.5 mm	0.106–0.25 mm	0.072–0.106 mm	<0.072 mm	
Duodenal digesta							
Diet 1 (*n* = 6) ^a^	1.30	6.58	18.33	10.31	3.51	59.98	181
Diet 2 (*n* = 6) ^a^	5.68	15.51	13.56	6.54	2.74	55.96	287
SEM	0.18	0.47	0.91	0.63	0.23	1.51	5
*p*-value	<0.01	<0.01	<0.01	<0.01	<0.01	0.04	<0.01
Ileal digesta							
Diet 1 (*n* = 6) ^a^	1.88	12.28	23.85	12.90	5.28	43.82	253
Diet 2 (*n* = 5) ^a^	5.35	20.26	16.85	8.95	3.84	44.77	331
SEM	0.08	0.39	0.70	0.49	0.26	1.74	6
*p*-value	<0.01	<0.01	<0.01	<0.01	<0.01	0.60	<0.01
Feces							
Diet 1 (*n* = 11) ^a^	1.09	8.05	21.42	8.07	2.72	58.65	195
Diet 2 (*n* = 11) ^a^	6.23	15.94	12.93	5.10	2.07	57.73	293
SEM	0.27	0.43	0.53	0.38	0.14	1.23	7
*p*-value	<0.01	<0.01	<0.01	<0.01	<0.01	0.46	<0.01

^a^*n* is the number of pigs per treatment. MPS, mean particle size; SEM, standard error of means.

**Table 4:** Effect of dietary particle size on the digestive enzyme activities in duodenal fluid and digestibility of dietary nutrients in pigs (DM basis) ^a^.

Item	Diet 1	Diet 2	SEM	*p*-Value
Digestive enzyme activity of duodenal fluid, U/mL				
Amylase	147.7	137.6	33.5	0.77
Trypsin	43.6	46.0	5.8	0.68
Chymotrypsin	4.7	7.2	1.2	0.07
Apparent digestibility of nutrients, % ^b^				
ATTD of DM	88.6	88.2	0.4	0.26
ATTD of CP, %	86.6	86.4	0.3	0.55
ATTD of EE, %	84.6	83.7	0.8	0.31
ATTD of NDF, %	60.3	57.9	1.9	0.24
ATTD of ADF, %	54.7	59.5	3.0	0.15
ATTD of GE, %	88.8	88.5	0.4	0.43
DE, kcal/kg	3970	3953	18	0.38

^a^ These values were determined with 6 pigs per diet. ^b^ ATTD, apparent total tract digestibility; ADF, acid detergent fiber; CP, crude protein; DM, dry matter; DE, digestible energy; EE, ether extract; GE, gross energy; NDF, neutral detergent fiber; SEM, standard error of means.

**Table 5:** Effect of particle size on in vitro digestibility ^a^ of DM, GE, and IVDE ^b^ in corn and diets (dry-matter basis).

Item	Digestibility of DM, %	Digestibility of GE, %	IVDE, kcal/kg
Corn			
1-mm sieve	82.5	82.7	3706
2-mm sieve	82.3	81.5	3641
SEM	0.4	0.5	23
*p*-value	0.52	0.07	0.03
Diet			
Diet 1	81.5	81.0	3574
Diet 2	81.2	80.6	3561
SEM	0.4	0.4	17
*p*-value	0.45	0.32	0.47

^a^ These values were mean of 4 replicates for each corn and 5 replicates for each diet. ^b^ IVDE, in vitro digestible energy; SEM, standard error of means.

## References

[1] Li Q.F., Zang J., Liu D.W., Piao X.S., Lai C.H., Li D.F. (2014). Predicting corn digestible and metabolizable energy content from its chemical composition in growing pigs. J. Anim. Sci. Biotechnol..

[2] Rojas O.J., Stein H.H. (2015). Effects of reducing the particle size of corn grain on the concentration of digestible and metabolizable energy and on the digestibility of energy and nutrients in corn grain fed to growing pigs. Livest. Sci..

[3] Lyu Z.Q., Li Q.F., Zhang S., Lai C.H., Huang C.F. (2019). Available energy and amino acid digestibility of yellow dent corn fed to growing pigs. J. Anim. Sci..

[4] Lyu Z.Q., Li Q.F., Wu Y.F., Huang C.F. (2020). Effects of particle size and lipid form of corn on energy and nutrient digestibility in diets for growing pigs. Asian Australas. J. Anim. Sci..

[5] Healy B.J., Hancock J.D., Kennedy G.A., Bramel-Cox P.J., Behnkes K.C., Hines R.H. (1994). Optimum particle size of corn and hard and soft sorghum for nursery pigs. J. Anim. Sci..

[6] Hancock J.D., Behnke K.C., Lewis A.J., Southern L.L. (2001). Use of ingredient and diet processing technologies (grinding, mixing, pelleting, and extruding) to produce quality feeds for pigs. Swine Nutrition.

[7] Al-Rabadi G.J., Hosking B.J., Torley P.J., Williams B.A., Bryden W.L., Nielsen S.G., Black J.L., Gidley M.J. (2017). Regrinding large particles from milled grains improves growth performance of pigs. Anim. Feed Sci. Technol..

[8] Amaral N.O., Amaral L.G.M., Cantarelli V.S., Fialho E.T., Zangeronimo M.G., Rodrigues P.B. (2015). Influence of maize particle size on the kinetics of starch digestion in the small intestine of growing pigs. Anim. Prod. Sci..

[9] Wondra K.J., Hancock J.D., Behnke K.C., Hines R.H. (1995). Effects of particle size and pelleting on growth performance, nutrient digestibility, and stomach morphology in finishing pigs. J. Anim. Sci..

[10] Huang C., Zang J., Song P., Fan P., Chen J., Liu D., He P., Ma X. (2015). Effects of particle size and drying methods of corn on growth performance, digestibility and haematological and immunological characteristics of weaned piglets. Arch. Anim. Nutr..

[11] Vukmirović Đ., Čolović R., Rakita S., Brlek T., Đuragić O., Solà-Oriol D. (2017). Importance of feed structure (particle size) and feed form (mash vs. pellets) in pig nutrition. Anim. Feed Sci. Technol..

[12] Grundy M.M., Edwards C.H., Mackie A.R., Gidley M.J., Butterworth P.J., Ellis P.R. (2016). Re-evaluation of the mechanisms of dietary fibre and implications for macronutrient bioaccessibility, digestion and postprandial metabolism. Br. J. Nutr..

[13] Saqui-Salces M., Luo Z., Urriola P.E., Kerr B.J., Shurson G.C. (2017). Effect of dietary fiber and diet particle size on nutrient digestibility and gastrointestinal secretory function in growing pigs. J. Anim. Sci..

[14] Dang F.K. (2018). Study on Purification of Digestive Enzymes in Intestinal Tract and In Vitro Simulation of Intestinal Fluid for Growing Pigs. Master’s Thesis.

[15] NRC (2012). Nutrient Requirements of Swine.

[16] Zhao F., Hou S.S., Zhang H.F., Zhang Z.Y. (2007). Effect of dietary metabolizable energy and crude protein content on the activities of digestive enzymes in jejunal fluid of pekingduck. Poult. Sci..

[17] Lentle R.G., Ravindran V., Ravindran G., Thomas D.V. (2006). Influence of feed particle size on the efficiency of broiler chickens fed wheat-based diets. J. Poult. Sci..

[18] Gao Q.T., Zhao F., Zhang H., Wang Y. (2019). Determination of the particle size for pelleted diet, digesta and feces of pig using wet-sieving method. Acta Veterinaria et Zootechnica Sinica.

[19] Dahlqvist A. (1962). A method for the determination of amylase in intestinal content. Scand. J. Clin. Lab. Investig..

[20] Wirnt R., Bergmeyer H.U. (1974). Trypsin, measurement with n-α-p-toluenesulfonyl-l-arginine methyl ester as substrate. Methods of Enzymatic Analysis.

[21] Wirnt R., Bergmeyer H.U. (1974). Chymotrypsin, measurements with n-benzoyl-l-tyrosin methyl ester as substrate. Methods of Enzymatic Analysis.

[22] Kong C., Adeola O. (2014). Evaluation of amino Acid and energy utilization in feedstuff for Swine and poultry diets. Asian Australas. J. Anim. Sci..

[23] Gao Q.T. (2019). Study on Determination of Effective Energy in Cereal Grains Based on Simulating In Vivo Digestion Process for Growing Pigs. Master’s Thesis.

[24] AOAC (2007). Official Methods of Analysis.

[25] Van Soest P.J., Robertson J.B., Lewis B.A. (1991). Methods for dietary fiber, neutral detergent fiber, and nonstarch polysaccharides in relation to animal nutrition. J. Dairy Sci..

[26] Clauss M., Fritz J., Tschuor A., Braun U., Hummel J., Codron D. (2017). Dry matter and digesta particle size gradients along the goat digestive tract on grass and browse diets. J. Anim. Physiol. Anim. Nutr..

[27] Adeola O., Lewis A., Southern L., Lewis A.J., Southern L.L. (2001). Digestion and balance techniques in pigs. Swine Nutrition.

[28] Zhao F., Zhang L., Mi B.M., Zhang H.F., Hou S.S., Zhang Z.Y. (2014). Using a computer-controlled simulated digestion system to predict the energetic value of corn for ducks. Poult. Sci..

[29] SAS (2004). The BOXPLOT Procedure. SAS/STAT 9.1 User’s Guide.

[30] Wilfart A., Montagne L., Simmins P.H., Van Milgen J., Noblet J. (2007). Sites of nutrient digestion in growing pigs: Effect of dietary fiber. J. Anim. Sci..

[31] Regina D.C., Eisemann J.H., Lang J.A., Argenzio R.A. (1999). Changes in gastric contents in pigs fed a finely ground and pelleted or coarsely ground meal diet. J. Anim. Sci..

[32] Parsons A.S., Buchanan N.P., Blemings K.P., Wilson M.E., Moritz J.S. (2006). Effect of corn particle size and pellet texture on broiler performance in the growing phase. J. Appl. Poult. Res..

[33] Jankowski J., Mikulski D. (2013). A note on the particle size distribution of intestinal digesta and nutrient digestibility in growing turkeys fed diets with different whole-grain wheat contents. J. Anim. Feed Sci..

[34] Maulfair D., Fustini M., Heinrichs A. (2011). Effect of varying total mixed ration particle size on rumen digesta and fecal particle size and digestibility in lactating dairy cows. J. Dairy Sci..

[35] Liu C.L., Zhao F., Zuo J.J., Wang Y.M., Zhang L., Gao L.X., Zhang H.F. (2014). Variation of nutrients flow and recovery rate of inert marker in digesta collected with T-cannula at the terminal ileum of growing pigs. Chin. J. Anim. Nutr..

[36] Wang Y.M., Zhao F., Liao R., Zhang L., Zhang H., Zhang H.F. (2015). Correlation study between digestive enzyme activity of jejunal fluid and nutrient digestibility of diets in growing pigs. Chin. J. Anim. Nutr..

[37] Partridge I.G., Low A.G., Sambrook I.E., Corring T. (1982). The influence of diet on the exocrine pancreatic secretion of growing pigs. Br. J. Nutr..

[38] Zebrowska T., Low A.G. (1987). The influence of diets based on whole wheat, wheat flour and wheat bran on exocrine pancreatic secretion in pigs. J. Nutr..

[39] Wondra K.J., Hancock J.D., Behnke K.C., Stark C.R. (1995). Effects of mill type and particle size uniformity on growth performance nutrient digestibility and stomach morphology in finishing pigs. J. Anim. Sci..

[40] Kim I.H., Hancock J.D., Hong J.W., Cabrera M.R., Hines R.H., Behnke K.C. (2002). Corn particle size affects nutritional value of simple and complex diets for nursery pigs and broiler chicks. Asian Australas. J. Anim. Sci..

[41] Bao Z., Li Y., Zhang J., Li L., Zhang P., Huang F.R. (2016). Effect of particle size of wheat on nutrient digestibility, growth performance, and gut microbiota in growing pigs. Livest. Sci..

[42] Paulk C.B., Hancock J.D., Fahrenholz A.C., Wilson J.M., Mckinny L.J., Behnke K.C. (2015). Effects of sorghum particle size on milling characteristics and growth performance in finishing pigs. Anim. Feed Sci. Technol..

[43] Liu P., Souza L.W.O., Baidoo S.K., Shurson G.C. (2012). Impact of distillers dried grains with solubles particle size on nutrient digestibility, DE and ME content, and flowability in diets for growing pigs. J. Anim. Sci..

